# Reply to Halstead and Sayce et al

**DOI:** 10.1093/cid/cis1049

**Published:** 2013-03-15

**Authors:** Dong Thi Hoai Tam, Cameron P. Simmons, Bridget A. Wills

**Affiliations:** 1 Department of Infectious Diseases, University of Medicine and Pharmacy of Ho Chi Minh City; 2 Centre for Tropical Medicine, Oxford University Clinical Research Unit, Ho Chi Minh City, Vietnam

To the Editor—We agree with Dr Halstead that the findings of our trial of early corticosteroid therapy in Vietnamese children and young adults with dengue add an interesting perspective to the long-running debate on the mechanisms underlying disease pathogenesis [1, 2]. The absence of any adverse effects of prednisolone therapy on the virological safety parameters assessed, together with the lack of a reduction in the frequency of shock or other complications, brings into question the concept of an immunopathogenic storm being causally responsible for the microvascular dysfunction. However, we caution against overinterpreting the clinical trial results as representing definitive evidence against a role for T cells or any other immunological mechanisms. Instead, we believe our results emphasize how little we understand about the disease processes responsible for these complications and hope that these results will trigger more expansive efforts in dengue pathogenesis research.

Detailed examination of serial measurements of immunological events in study participants will be published elsewhere (manuscript in preparation), but it is clear that the pharmacological “footprint” of prednisolone therapy in this trial was less than anticipated. Perhaps earlier therapy, or higher treatment doses, might have elicited a greater effect on clinical and laboratory end points, but given the dysglycemia encountered with the 2 mg/kg dosage regimen, the latter option would not be feasible for large-scale intervention in the community.

The potential role of dengue nonstructural protein 1 (NS1) in pathogenesis is an important area to consider, but is not without difficulties. Importantly, there are conflicting data on the relationship between concentrations of secreted NS1, plasma viremia, immune status, and clinical disease severity [3–5]. In particular, plasma levels of dengue virus 2–associated NS1 are often low or undetectable in both human studies and mouse models [4, 6], yet dengue virus 2 is well established as a cause of severe disease. Secondly, kinetic studies reveal that although plasma levels of NS1 are often high in early disease, the protein may also persist for several weeks after infection without causing clinical complications [7], suggesting that if NS1 is important in pathogenesis, other factors must also be operating to influence outcome. We agree that complement activation, possibly exacerbated by NS1, is probably important and is a relatively poorly researched area of dengue pathogenesis.

Sayce and colleagues describe data from a series of experiments that investigate dexamethasone as a dengue therapeutic in primary monocyte-derived macrophages from dengue-naive donors [8]. They observed a significant but transient decrease in viral load on day 1 with dexamethasone treatment, concomitant with a relative reduction in levels of selected inflammatory cytokines, and they comment that the effectiveness of treatment with steroids may depend critically on time of drug administration. However, interpretation of these results in terms of overall disease pathogenesis is difficult, because the true relevance of isolated cell culture systems to human disease processes is doubtful.

The idea of combined antiviral and immunomodulatory therapy is certainly one avenue that may be worth pursuing, particularly if it is possible to initiate the combined therapy very early in the disease evolution. Efforts directed toward improving dengue rapid diagnostics suitable for use early in the febrile phase, together with research to identify risk factors associated with subsequent progression to severe disease, are important if this strategy is to be considered. In the end, if a simple, safe, and effective therapy or combination of therapies does become available, it is crucial that prompt intervention targeted toward high-risk groups is a realistic possibility. Finally, although the mechanisms underlying microvascular dysfunction in dengue are almost certainly multifactorial, it is hoped that the recent momentum in clinical intervention trials will eventually lead to improved treatment options for patients with dengue as well as insights into pathogenesis [2, 7, 9, 10].
